# The complex formation of MASP-3 with pattern recognition molecules of the lectin complement pathway retains MASP-3 in the circulation

**DOI:** 10.3389/fimmu.2022.907023

**Published:** 2022-08-16

**Authors:** Kohei Kusakari, Takeshi Machida, Yumi Ishida, Tomoko Omori, Toshiyuki Suzuki, Masayuki Sekimata, Ikuo Wada, Teizo Fujita, Hideharu Sekine

**Affiliations:** ^1^ Department of Immunology, Fukushima Medical University, Fukushima, Japan; ^2^ Radioisotope Research Center, Fukushima Medical University, Fukushima, Japan; ^3^ Department of Cell Science, Institute of Biomedical Sciences, Fukushima Medical University, Fukushima, Japan; ^4^ Fukushima Prefectural General Hygiene Institute, Fukushima, Japan

**Keywords:** complement, MASP-3, alternative pathway, lectin pathway, pattern recognition molecules

## Abstract

The complement system plays an important role in host defense and is activated *via* three different activation pathways. We have previously reported that mannose-binding lectin-associated serine protease (MASP)-3, unlike its splicing variant MASP-1, circulates in an active form and is essential for the activation of the alternative pathway (AP) *via* the activation of complement factor D (FD). On the other hand, like MASP-1 and MASP-2 of the lectin pathway (LP), MASP-3 forms a complex with the pattern recognition molecules (PRMs) of the LP (LP-PRMs). Both MASP-1 and MASP-2 can be activated efficiently when the LP-PRMs complexed with them bind to their ligands. On the other hand, it remains unclear how MASP-3 is activated, or whether complex formation of MASP-3 with LP-PRMs is involved in activation of MASP-3 or its efficiency in the circulation. To address these issues, we generated wild-type (WT) and four mutant recombinant mouse MASP-3 proteins fused with PA (human podoplanin dodecapeptide)-tag (rmMASP-3-PAs), the latter of which have single amino acid substitution for alanine in the CUB1 or CUB2 domain responsible for binding to LP-PRMs. The mutant rmMASP-3-PAs showed significantly reduced *in-vivo* complex formation with LP-PRMs when compared with WT rmMASP-3-PA. In the *in-vivo* kinetic analysis of MASP-3 activation, both WT and mutant rmMASP-3-PAs were cleaved into the active forms as early as 30 minutes in the circulation of mice, and no significant difference in the efficiency of MASP-3 cleavage was observed throughout an observation period of 48 hours after intravenous administration. All sera collected 3 hours after administration of each rmMASP-3-PA showed full restoration of the active FD and AP activity in MASP-3-deficient mouse sera at the same levels as WT mouse sera. Unexpectedly, all mutant rmMASP-3-PAs showed faster clearance from the circulation than the WT rmMASP-3-PA. To our knowledge, the current study is the first to show *in-vivo* kinetics of MASP-3 demonstrating rapid activation and clearance in the circulation. In conclusion, our results demonstrated that the complex formation of MASP-3 with LP-PRMs is not required for *in-vivo* activation of MASP-3 or its efficiency, but may contribute to the long-term retention of MASP-3 in the circulation.

## Introduction

The complement system plays essential roles in the innate immunity (1). It is activated *via* three different activation pathways; the classical pathway (CP), lectin pathway (LP), and alternative pathway (AP) (2). Activations of the CP and LP are initiated by the bindings of pattern recognition molecules (PRMs) to their ligands, i.e., binding of C1q to antigen-antibody complexes in the CP activation and binding of mannose-binding lectin (MBL), ficolins, collectin liver 1 (CL-L1 or CL-10), collectin kidney 1 (CL-K1 or CL-11) or their heterocomplex CL-LK to carbohydrates in the LP activation. Those PRMs circulate in a complex with serine proteases, C1r and C1s in the CP, and MBL-associated serine protease (MASP)-1 and MASP-2 in the LP. At the site of PRM-binding, the primary serine proteases, C1r (3) or MASP-1 (4), complexed therewith autoactivated and in turn activate the secondary serine proteases, C1s or MASP-2 (5). Afterwards, those activated secondary serine proteases cleave complement components C4 and C2 to generate a CP/LP C3 convertase, C4b2a. Eventually, C4b2a cleaves C3 into C3a and C3b; the former serves as an anaphylatoxin, and the latter may covalently bind to proteins or carbohydrates on microbial surfaces or host tissues.

On the other hand, the AP has no PRMs, and its activation is initiated by the low-level spontaneous hydrolysis of C3 on the bacterial surfaces to generate C3(H_2_O) (6). Once C3(H_2_O) is generated, complement factor B (FB) binds to C3(H_2_O), and is cleaved into Ba and Bb fragments by an active form of complement factor D (FD), which is already activated by MASP-3, to generate an initial AP C3 convertase, C3(H_2_O)Bb, which cleaves C3 to generate C3b that is bound covalently to pathogens. In turn, FB forms a complex with C3b and is cleaved by active FD, leading to the formation of the AP C3 convertase, C3bBb. In this way, the AP enhances complement activation at the C3b-binding site *via* the amplification loop. Unlike C1r, C1s, MASP-1, and MASP-2 in the CP and LP, the serine proteases MASP-3 and FD circulate in the active forms both in humans and mice (7–9); however, the activation mechanism of MASP-3 remains unclear.

MASP-3 is a splicing variant of MASP-1, and both are transcribed from the *MASP1* gene (10). They have a common heavy chain (H-chain) consisting of five domains, in the following order: an N-terminal C1r/C1s/Uegf/bone morphogenetic protein (CUB1) domain; an epidermal growth factor (EGF)-like domain; a secondary CUB (CUB2) domain; a primary complement control protein (CCP1) domain; and a secondary CCP (CCP2) domain. Since the CUB1-EGF-CUB2 motif is responsible for complex formation with the LP-PRMs, such as MBL, ficolins, CL-L1, CL-K1 and CL-LK, both MASP-1 and MASP-3 can form a complex with LP-PRMs. Some evidence that MASP-3 forms a complex with LP-PRMs has been reported. MASP-3 was first isolated as a complex with MBL from human plasma (10). Skjoedt et al. (11) reported that the mean concentration of MASP-3 was 6.4 mg/L in human serum, and a large portion of MASP-3 circulated in a complex with ficolin-3 rather than MBL or ficolin-2. Teillet et al. (12) have reported that, using surface plasmon resonance analysis, recombinant human MASP-3 with a single amino acid substitution at the CUB1 or CUB2 domain including E49, D102, H218, and Y225 for alanine shows significantly reduced ability to associate with LP-PRMs such as human MBL, L-ficolin (ficolin-2), and H-ficolin (ficolin-3). Afterwards, Henriksen et al. (13) reported that CL-LK formed a complex with MASPs including MASP-3 and could mediate LP activation. When MASP-3 was first discovered, its role in the complex with LP-PRM was thought to be a down-regulator of LP activation by displacement and/or competition with MASP-2 (10). However, our recent studies have shown that MASP-3-deficient mouse sera have comparable LP activity to WT mouse sera (9). Therefore, our results suggest that MASP-3 in complex with LP-PRMs is not involved in the activation/regulation of the LP *in vivo*.

In contrast to the H-chains common to MASP-1 and MASP-3, their light chains (L-chains) consisting of a serine protease domain are transcribed from different exons of the *MASP1* gene, and their structure and functions are different (14–17). Indeed, we have recently reported that MASP-1 and MASP-3 play independent roles in physiological activations of the LP and AP, respectively (9). MASP-1 and MASP-2 of the LP circulate in proenzyme forms as do C1r and C1s of the CP. When these serine proteases are activated, C1 inhibitor (serpin G1) acts as their pseudosubstrate and irreversibly inhibits their activity (18, 19). On the other hand, MASP-3, which circulates mostly in an active form, has no known physiological inhibitors, and its activation or clearance kinetics *in vivo* are largely unknown.

MASP-3 has been shown to play an important role not only in complement activation *via* activation of FD, but also in embryonic development. Loss-of-function of MASP-3 due to congenital mutations in MASP-3-specific exon of the *MASP1* gene results in human 3MC syndrome characterized by a spectrum of developmental features, including developmental delay, growth retardation, intellectual disability, and characteristic facial atypia (20). Of interest, congenital mutations in the CL-L1 or CL-K1 genes also cause 3MC syndrome (21, 22). In case of mice, we have previously reported significantly reduced body weight in the MASP-3-deficient mice compared with the WT littermates (9). Taken together, it can be inferred that MASP-3 plays an important role in embryonic development by forming a complex with CL-L1, CL-K1 or CL-LK. However, the role or functional benefits of MASP-3 forming a complex with LP-PRMs in complement activation are largely unclear.

In the current study, we hypothesized that complex formation of MASP-3 with LP-PRMs is involved in the activation of MASP-3 in the circulation, similarly to the activation of MASP-1. To clarify this hypothesis, we generated a recombinant wild-type and four mutant mouse MASP-3 proteins with E49A, D102A, H218A or Y225A mutation in the CUB1 or CUB2 domain; these mutations significantly decreased the ability of human MASP-3 to associate with MBL, L-ficolin, and H-ficolin (12). These recombinant mouse MASP-3 proteins were administered to wild-type and MASP-3-deficient mice, and their activation and clearance kinetics in the circulation were analyzed.

## Materials and methods

### Mice

Wild-type C57BL/6J mice (C57BL/6JJcl) were purchased from CLEA Japan, Inc. (Tokyo, Japan). MASP-3-deficient C57BL/6J mice were generated by genome editing using CRISPR/Cas9 system in our previous study (9) and bred in-house for use in the current study. The WT or MASP-3-deficient C57BL/6J mice used were aged 8−14 weeks. All animal experiments, including housing, breeding, and use of the mice, were reviewed and approved by the Animal Experiments Committee of Fukushima Medical University (approval no. 2021012) and performed in accordance with the guidelines for the care and use of laboratory animals established by the committee.

### Plasmid construction

A recombinant WT mouse MASP-3 protein was generated as a fusion protein with a PA-tag, a dodecapeptide derived from human podoplanin (23), at the C-terminus, termed WT rmMASP-3-PA. A full-length coding sequence of mouse MASP-3 was amplified by PCR using primers designed based on the full-length cDNA sequence of mouse MASP-3 (GenBank accession no. AB049755). The amplified cDNA fragment was introduced into a pCAG-Bsd PA tag-C vector (Wako, Osaka, Japan) using the In-Fusion^®^ HD Cloning Kit (Takara Bio, Kyoto, Japan) according to the manufacturer’s instructions, and the construct was transformed into *Escherichia coli* DH5α to amplify the plasmid.

**Figure 1:**
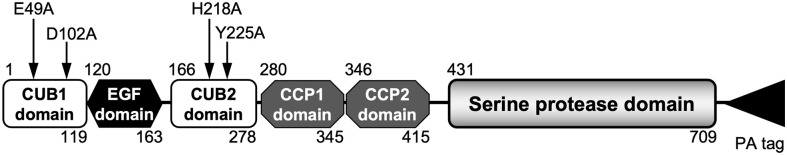
A schematic domain structure of rmMASP-3-PA. The numbers at the top and bottom represent the first and last amino acid numbers in each domain, respectively according to the information in the UniProt database (UniProt ID: P98064). The arrows indicate the four different positions of the single amino acid substitutions for alanine used in the current study.

Four different mutant rmMASP-3s, which have single amino acid substitution for alanine at E49 (E49A), D102 (D102A), H218 (H218A) or Y225 (Y225A), as depicted in **Figure 1**, were generated using the pCAG-Bsd PA tag-C/WT rmMASP-3 plasmid and a PrimeSTAR Mutagenesis Basal Kit (Takara Bio) according to the manufacturer’s instructions. The primers used for amplification of mutant rmMASP-3 cDNAs were: 5’- aacttgGCCtcctcctatctttgtgaa -3’ and 5’- ggaggaGGCcaagttgaagtgcatga -3’ for the E49A mutant; 5’- cggtcaGCTttctccaatgaggaacg -3’ and 5’- ggagaaAGCtgaccggaaagtgacag -3’ for the D102A mutant; 5’- tgaagacGCTcctgaggtgccctgtcc -3’ and 5’- tcaggAGCgtcttcaatgtcaaaaat -3’ for the H218A mutant; and 5’- ctgtcccGCTgactacattaagattaa -3’ and 5’- tagtcAGCgggacagggcacctcagg -3’ for the Y225A mutant. The codon for the substituted amino acid was in capital letters. The mutant DNA products were introduced into the pCAG-Bsd PA tag-C vector and then transformed into *E. coli*, as in the case of WT rmMASP-3.

Another set of WT rmMASP-3 protein was generated as an ALFA-tagged protein (24) in its C-terminus, termed WT rmMASP-3-ALFA. A full-length coding sequence of mouse MASP-3 was amplified by PCR using primers containing nucleotides corresponding to ALFA tag and an additional proline residue between them, which acts as an insulator (-PSRLEEELRRRLTE). The amplified cDNA fragment was introduced into a pCAG-Bsd PA tag-C vector and transformed into *E. coli*. Introduction of the objective cDNA fragment was confirmed by DNA sequencing.

### Protein expression and purification

Plasmids for expression of WT or mutant rmMASP-3-PAs and WT rmMASP-3-ALFA were transfected into Chinese hamster ovary (CHO) cells with the FuGene-HD transfection reagent (Roche, Indianapolis, IN, USA) according to the manufacturer’s instructions. After transfection, cells that were resistant to blastcidin S (Wako) were transferred to EX-CELL^®^ 325 PF CHO serum-free medium (Sigma-Aldrich, St Louis, MO, USA) for efficient expression of introduced genes. Culture supernatant containing expressed protein was collected and subjected to purification using anti-PA tag antibody beads (Wako) for PA-tagged proteins or ALFA Selector ST (NanoTag Biotechnologies, Göttingen, Germany) for ALFA-tagged proteins. The rmMASP-3-PAs or rmMASP-3-ALFA bound to the beads were eluted with glycine-HCl buffer (pH 2.5) followed by addition of 1/10 volume of 1 M Tris-HCl (pH 9.0) for neutralization. Expression and purification of target proteins were checked by SDS-PAGE under reducing condition followed by staining with InstantBlue staining solution (Expedeon, Heidelberg, Germany) or by Western blotting using horseradish peroxidase (HRP)-conjugated anti-mouse MASP-3 L-chain antibody (9). After the blotted membrane was treated with ECL Prime Western Blotting Detection System (GE Healthcare, Buckinghamshire, UK), objective protein bands were visualized by chemiluminescence detection using an Amersham Imager 600 (GE Healthcare). To confirm the mutation of the proteins, the purified samples were subjected to mass spectrometry according to the method reported by Takahashi et al. (16).

### Assay for *in-vivo* complex formation of rmMASP-3-PA with LP-PRMs

One-hundred micrograms of each of the rmMASP-3-PAs dissolved in PBS were administered to the tail vein of the MASP-3-deficient C57BL/6J mice. The mice were bled 3 h after administration. Serum LP-PRMs that formed complexes with the administered rmMASP-3-PA were isolated by immunoprecipitation using anti-PA tag antibody beads. Briefly, 100 µL of serum samples were mixed with 50 µL of anti-PA antibody beads in a total of 200 µL with TBS supplemented with 7 mM MgCl_2_ and 5 mM CaCl_2_ (TBS/Mg/Ca), and then the mixture was incubated at 4°C overnight with inverted mixing. After centrifugation, the precipitated beads were washed four times with TBS/Mg/Ca and then resuspended in SDS-PAGE sample buffer with 2-mercaptoethanol in a volume equivalent to serum followed by incubation at 80°C for 10 min. Sera of mice administered with WT rmMASP-3-PA was separately subjected to immunoprecipitation using TBS supplemented with 7 mM MgCl_2_ and 10 mM EGTA (TBS/Mg/EGTA) as a negative control in which the rmMASP-3-PA/LP-PRM complex is dissociated.

The eluates were subjected to SDS-PAGE under reducing condition followed by Western blotting. The blotted membranes were separately subjected to immunodetection using following primary antibodies; HRP-conjugated rat anti-mouse MBL-A monoclonal antibody (clone: 8G6, Hycult Biotech, Plymouth Meeting, PA, USA), HRP-conjugated rat anti-mouse MBL-C monoclonal antibody (clone: 16A8, Hycult Biotech), rabbit anti-ficolin-A polyclonal antibody (25), and rabbit anti-CL-K1 polyclonal antibody (Proteintech, Rosemont, IL, USA), followed by reaction with a HRP-conjugated secondary antibody for rabbit first antibodies. After the membranes were treated with ECL Prime Western Blotting Detection System, objective protein bands were visualized by chemiluminescence detection using the Amersham Imager 600. The band intensity detected was measured using an ImageQuant TL software (GE healthcare). For membrane stripping, the membranes were washed twice with PBS containing 0.5% Tween-20 (PBST) for 5 min and then incubated in Stripping solution (Wako) for 10 min at room temperature with shaking. After washing three times with PBST, the membranes were used for another detection. Immunodetection was also performed using HRP-conjugated anti-PA tag antibody to normalize the loading amount of rmMASP-3-PA between samples.

### *In-vivo* activation of rmMASP-3-PA

One-hundred micrograms of each of the rmMASP-3-PAs dissolved in PBS were administered to the tail vein of the WT or MASP-3-deficient C57BL/6J mice. The mice were bled before rmMASP-3-PA administration and at 0.5, 1.5, 3, 6, 12, 24, 48 h after administration. The serum samples were collected and subjected to SDS-PAGE under reducing condition followed by Western blotting using the HRP-conjugated anti-PA tag antibody and ECL Prime Western Blotting Detection System. After photographed by the chemiluminescence mode using an Amersham Imager 600, the band intensity detected was measured using an ImageQuant TL software.

### Western blotting of FD

Mouse FD in the serum samples of MASP-3-deficient C57BL/6J mice obtained 3 h after administration of rmMASP-3-PA was immunoprecipitated with rabbit anti-mouse FD antibody, deglycosylated, and subjected to Western blotting according to the method described previously (16). Notably, mouse serum FD is detected as a smeared band by Western blotting due to various glycosylation patterns when it is not deglycosylated (data not shown). The difference in molecular mass between mouse pro-FD (26.1 kDa) and active FD (25.5 kDa) is only 0.6 kDa. In order to detect the difference by Western blotting, the immunoprecipitated serum FD was deglycosylated with N-glycosidase F (Merck, Darmstadt, Germany) and then analyzed by Western blotting.

### C3 deposition assay on zymosan

The AP activity of serum samples from the MASP-3-deficient C57BL/6J mice obtained 3 h after administration of rmMASP-3-PA was measured using 10% sera diluted with TBS/Mg/EGTA and zymosan-coated microplates according to the method described previously (16). C3 deposition level on zymosan was detected with HRP-conjugated anti-PA tag antibody followed by color development with TMB substrate solution (Kirkegaard & Perry Laboratories, Gaithersburg, MD, USA). The absorbance at 450 nm was measured by a Varioskan LUX multimode microplate reader (Thermo Fisher Scientific).

### Homodimer formation of MASP-3

Homodimer formation of rmMASP-3 was examined using WT/mutant rmMASP-3-PAs and WT rmMASP-3-ALFA based on the method described by Rosbjerg et al. (26). An equivalent amount (10 µg) of each rmMASP-3-PA and rmMASP-3-ALFA was mixed and dialyzed against TBS containing 10 mM EDTA (TBS/EDTA) for 24 h at 4°C. An aliquot of the dialyzed sample (4 µg of each rmMASP-3) was further dialyzed against TBS containing 5 mM CaCl_2_ (TBS/Ca) for 24 h at 4°C and then adjusted with 20% Blocking One in TBS/Ca to 700 µL (0.14 µM of each rmMASP-3). Another aliquot was placed on ice for 24 h and then adjusted with 20% Blocking One in TBS/EDTA to 700 µL (0.14 µM of each rmMASP-3). Those samples were serially diluted with 20% Blocking One in TBS/Ca or TBS/EDTA as compatible to the buffer composition in which they dissolved, and then added to the microplate preliminarily coated with anti-PA tag antibody followed by 30-min incubation at room temperature. After washing the wells three times with PBST, rmMASP-3-ALFA that formed a homodimer with WT or mutant rmMASP-3-PAs was detected with HRP-conjugated anti-ALFA nanobody (NanoTag Biotechnologies) followed by color development with TMB substrate solution. The absorbance at 450 nm was measured using a Varioskan LUX multimode microplate reader.

### Statistical analysis

Statistical analysis was performed using a GraphPad Prism 8 software for Mac OS X (GraphPad Software, San Diego, CA, USA). Multiple groups were compared using a one-way ANOVA with *post-hoc* Tukey’s multiple comparison test.

## Results

### Generation of WT and mutant rmMASP-3-PAs

**Figure 2:**
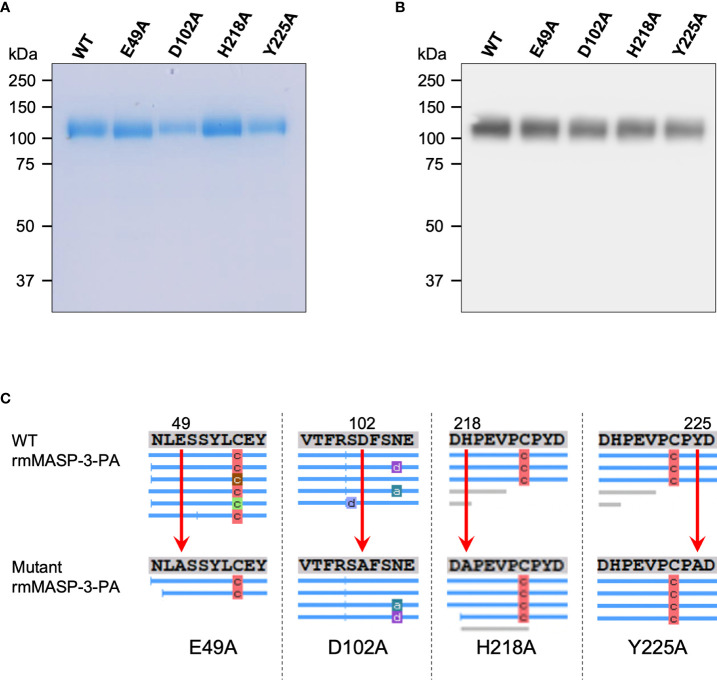
Expression and purification of WT and mutant rmMASP-3-PAs were confirmed by SDS-PAGE under reducing condition followed by InstantBlue staining **(A)** and Western blotting using anti-mouse MASP-3 L-chain antibody **(B)**. **(C)** The results on mass spectrometric analyses revealed the substitutions of target amino acids in rmMASP-3-PA (E49, D102, H218 or Y225) for alanine, as indicated by the red arrows. The blue lines below the amino acid sequences show the fragments detected by the mass spectrometry. The characters on the blue line represent as follows: “a” means ammonia-loss, “c” means carbamylation, and “d” means deamidation of the corresponding peptides in the peptide fragment, which are due to protease treatment prior to analysis with mass spectrometry.

To investigate our hypothesis that forming a complex with LP-PRMs is involved in the activation of MASP-3, we generated WT and mutant rmMASP-3 proteins (**Figure 1**). In the present study, the WT mouse MASP-3 cDNA and four different types of mutant mouse MASP-3 cDNAs were prepared by PCR and the site-directed mutagenesis, and then introduced into the pCAG-Bsd PA tag-C vector, to be expressed as PA-tagged proteins in their C-termini. The WT and mutant rmMASP-3-PA proteins were expressed in CHO cells and purified by an affinity chromatography using anti-PA tag antibody beads. Expression and purification of the proteins were confirmed by SDS-PAGE followed by InstantBlue staining and Western blotting. As shown in **Figure 2A**, the InstantBlue staining showed expression and purification of approximately 110-kDa proteins for all rmMASP-3-PAs. The Western blot analysis revealed that the 110-kDa bands in the lanes for all rmMASP-3-PA samples were detected with anti-mouse MASP-3 L-chain antibody (**Figure 2B**). Furthermore, the objective amino acid replacements in four mutant rmMASP-3-PAs were confirmed by mass spectrometry analysis (**Figure 2C**). Taking all of these results together, it was confirmed that the WT and mutant rmMASP-3-PAs were generated as we had designed.

### *In-vivo* complex formation of WT and mutant rmMASP-3-PAs with serum LP-PRMs

Teillet et al. (12) have reported that recombinant human MASP-3 with a single amino acid substitution at the positions including E49, D102, H218, and Y225 for alanine shows significantly reduced ability to associate with human ficolin-2, ficolin-3 and MBL. To confirm whether mutant rmMASP-3-PAs with single amino acid mutations, E49A, D102A, H218A or Y225A, show reduced ability to form a complex with mouse LP-PRMs *in vivo*, we isolated rmMASP-3-PA/LP-PRM complexes from MASP-3-deficient mouse sera obtained 3 h after administration of each rmMASP-3-PA by immunoprecipitation experiment using anti-PA tag antibody beads. The isolated fractions were subjected to reducing SDS-PAGE followed by Western blotting using antibodies against each mouse LP-PRM.

**Figure 3:**
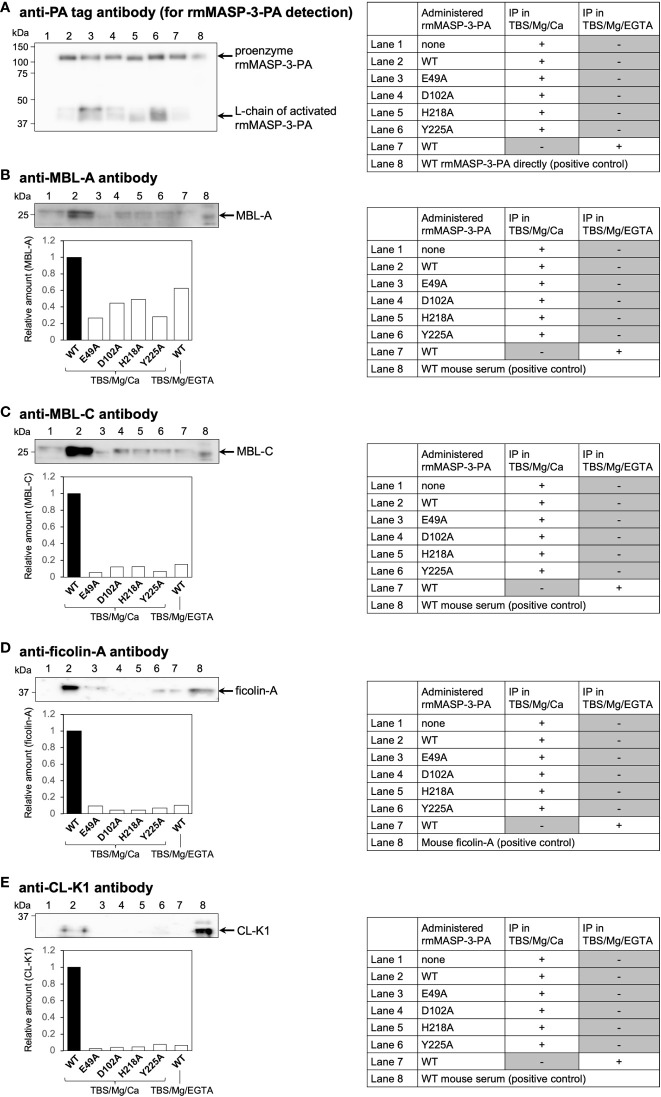
*In-vivo* complex formation of rmMASP-3-PA with endogenous MBL-A, MBL-C, ficolin-A or CL-K1. Immunoprecipitation (IP) experiments were performed using anti-PA tag antibody beads in TBS containing 7 mM MgCl_2_ and 5 mM CaCl_2_ (TBS/Mg/Ca) or 10 mM EGTA (TBS/Mg/EGTA). Each band intensity of MBL-A **(B)**, MBL-C **(C)**, ficolin-A **(D)**, and CL-K1 **(E)** was calibrated with the intensity of rmMASP-3-PA **(A)** obtained from the same IP sample to normalize the loading amount of rmMASP-3-PA between the samples. Relative amounts were expressed as the ratio of each calibrated intensity against that obtained from the mouse serum administered with WT rmMASP-3-PA (lower panels in **B–E**; WT = 1).

First, we analyzed rmMASP-3-PA levels in each immunoprecipitated fraction by Western blotting using anti-PA tag antibody. As shown in **Figure 3A**, the detection levels of WT or mutant rmMASP-3-PAs were different between the immunoprecipitated fractions. The band intensities of proenzyme and L-chain of activated rmMASP-3-PA were measured using an imaging software, and their sum was used to normalize total rmMASP-3-PA levels between the samples.

Next, we analyzed MBL-A (**Figure 3B**), MBL-C (**Figure 3C**), ficolin-A (**Figure 3D**), and CL-K1 (**Figure 3E**) levels in each immunoprecipitated fraction by Western blotting using antibodies specific for each LP-PRM. As shown in **Figures 3B–E**, Western blot analysis showed apparent detection levels of each LP-PRM in the immunoprecipitated fraction obtained from sera administered with WT rmMASP-3-PA (lane 2); the detection levels of that were significantly reduced in the immunoprecipitated fraction obtained in the presence of EGTA (lane 7). In contrast, Western blot analysis showed significantly reduced detection levels of each LP-PRM in the immunoprecipitated fraction obtained from sera administered with each mutant rmMASP-3-PA (lane 3–6) compared with that administered with WT rmMASP-3-PA (lane 2).

Detection of CL-L1 or CL-LK was not performed in the current study, since there was no commercially available antibody capable of detecting mouse CL-L1 by Western blotting. These results indicate that mutant rmMASP-3-PAs generated in the current study have little-to-no ability to form a complex with LP-PRMs, although it remains unclear whether they could form a complex with CL-L1 or CL-LK.

### *In-vivo* activation and clearance kinetics of WT and mutant rmMASP-3-PAs in the circulation of mice

**Figure 4:**
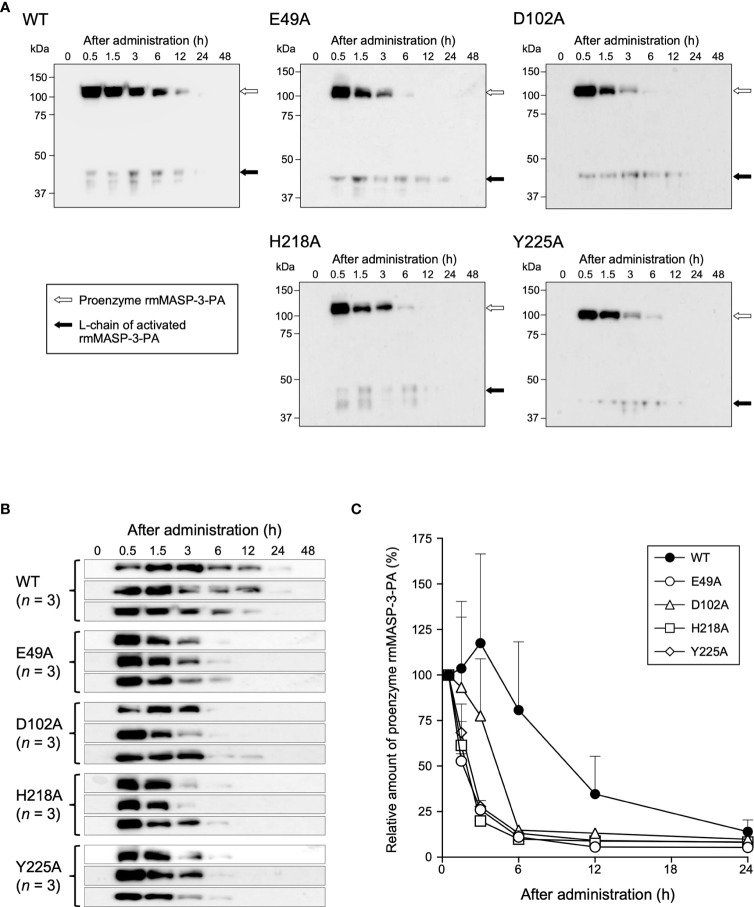
*In-vivo* activation and clearance kinetics of WT and mutant rmMASP-3-PAs when administered to mice. **(A)** Serum samples obtained at indicated time periods after administrations of rmMASP-3-PAs were subjected to SDS-PAGE under reducing condition followed by Western blotting using anti-PA tag antibody. The white and black arrows indicate bands for proenzyme rmMASP-3-PA and L-chain of activated rmMASP-3-PA, respectively. Similar results were obtained from three independent experiments, and representative images are shown here. **(B)** *In-vivo* clearance kinetics of proenzyme rmMASP-3-PAs when administered to mice (*n* = 3). **(C)** Residual amounts of the proenzyme rmMASP-3-PAs in the circulation are represented as the percentage of its band intensity against the intensity in the serum sample obtained 0.5 h after administration from the same mouse.

MASP-1 forms a complex with LP-PRMs in the circulation, which can bind to carbohydrates on microbial surfaces or damaged host tissues and trigger autoactivation of MASP-1. Next, we investigated whether the complex formation of MASP-3 with LP-PRMs is involved in the activation of MASP-3 in the circulation of MASP-3-deficient mice. Each 100 µg of rmMASP-3-PA was administered intravenously to MASP-3-deficient mice. Serum samples were then collected from the mice at 0.5, 1.5, 3, 6, 12, 24 and 48 h after administration, and analyzed by Western blotting using anti-PA tag antibody. As shown in **Figure 4A**, L-chains of cleaved MASP-3 (i.e., activated MASP-3) were detected in all sera collected 0.5 h after administration, suggesting that proenzyme MASP-3 is converted to the active form very rapidly in the circulation. In addition, the *in-vivo* kinetics analysis showed no significant difference in the efficiency of MASP-3 activation between WT and all mutant rmMASP-3-PAs. These results indicate that the complex formation of MASP-3 with LP-PRMs was not involved in MASP-3 activation in the circulation.

Unexpectedly, we observed different *in-vivo* clearance kinetics between the WT and mutant rmMASP-3-PAs. As shown in **Figures 4B, C**, the proenzyme WT rmMASP-3-PA was detected even 24 h after administration, whereas the proenzyme mutant rmMASP-3-PAs were no longer detected at 12 h after administration. The half-life of each rmMASP-3-PA in the circulation from 0.5 h after administration was calculated for each mouse. The half-life values (mean ± SD, *n* = 3) were 9.6 ± 4.9 h for WT, 1.2 ± 0.2 h for E49A, 3.2 ± 1.2 h for D102A, 1.3 ± 0.3 h for H218A, and 1.5 ± 0.3 h for Y225A. Thus, WT rmMASP-3-PA had a longer half-life than all mutant rmMASP-3-PAs.

These results suggest that complex formation of MASP-3 with LP-PRMs does not contribute to the activation of MASP-3, but does contribute to the long-term retention of MASP-3 in the circulation.

### Restoration of the active FD and AP activity in MASP-3-deficient mice by administration of rmMASP-3-PAs

**Figure 5:**
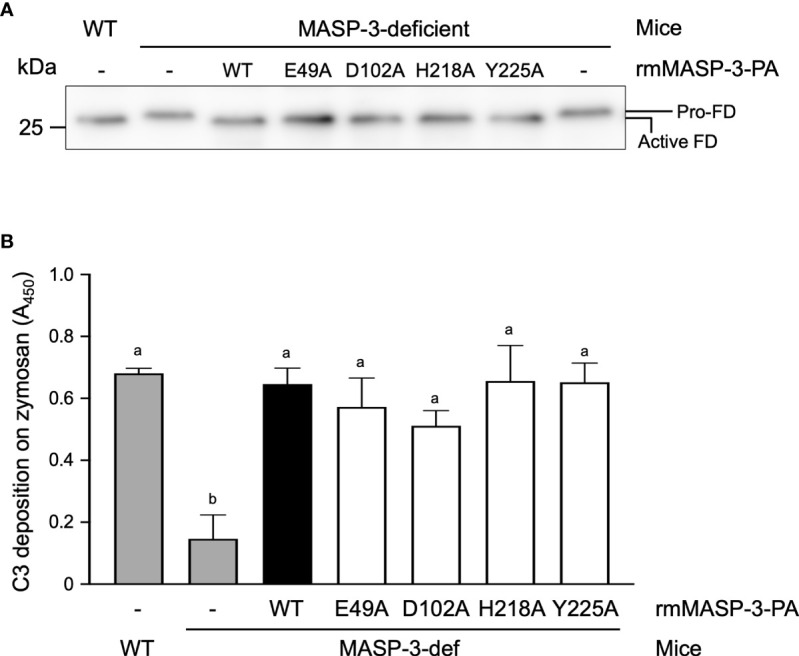
Restoration of the activation status of FD **(A)** and AP activity **(B)** in MASP-3-deficient mouse sera obtained 3 h after administrations of WT and mutant rmMASP-3-PAs. **(A)** Western blotting of FD was performed using serum samples that were preliminarily immunoprecipitated with anti-mouse FD antibody and then deglycosylated with N-glycosidase F. Pro-FD was detected at 26.1 kDa in MASP-3-deficient mouse sera (lane 2), while an active form FD was detected at a lower position (25.5 kDa; lane 1 and 3–7) than pro-FD. Similar results were obtained from three independent experiments, and a representative image is shown here. **(B)** The assay for the AP-driven serum C3 deposition activity was performed using a zymosan-coated microplate and TBS/Mg/EGTA under the Ca^2+^-chelated conditions. Values are means + SD (*n* = 3) obtained by subtracting the absorbance at 450 nm of a control sample without mouse serum from the absorbance at 450 nm of each sample. Values not sharing the same letters are significantly different at *P* < 0.05, as analyzed by *post-hoc* Tukey’s multiple comparisons.

We previously reported that sera from MASP-3-deficent mice had no or significantly reduced AP activity due to the lack of active FD in the circulation (9). We investigated whether the active FD and AP activity in MASP-3-deficient mouse could be restored by intravenous administration of rmMASP-3-PAs. First, sera collected 3 h after administration of WT or mutant rmMASP-3-PA were applied to Western blotting of FD to analyze the activation status of FD. As shown in **Figure 5A**, FD was detected as a 26.1 kDa protein in the sera of MASP-3-deficient mice collected prior to administration of rmMASP-3-PAs. On the other hand, FD was detected as a 25.5 kDa protein in the sera of MASP-3-deficient mice administered with each rmMASP-3-PA. These results indicate that most of the circulating proenzyme form of FD in the MASP-3-deficient mice was converted to the active form 3 h after administration of WT and mutant rmMASP-3-PAs.

Next, the sera collected above were subjected to C3 deposition assay on a zymosan-coated microplate to assess the AP activity. As shown in **Figure 5B**, sera from MASP-3-deficient mice administered with WT or mutant rmMASP-3-PAs showed a significant increase in C3 deposition levels compared to that prior to administration. There was no significant difference in C3 deposition levels between the sera from WT mice and those from MASP-3-deficient mice administered with each rmMASP-3-PA. These results indicate that the serum AP activity of MASP-3-deficient mice was restored to the same level as that of WT mice 3 h after administration of WT and mutant rmMASP-3-PAs.

Taken together, these results suggest that WT and the four mutant rmMASP-3-PAs can equally restore active FD and AP activity in the circulation when administered to MASP-3-deficient mice.

### *In-vitro* dimer formation of WT and mutant rmMASP-3-PAs

**Figure 6:**
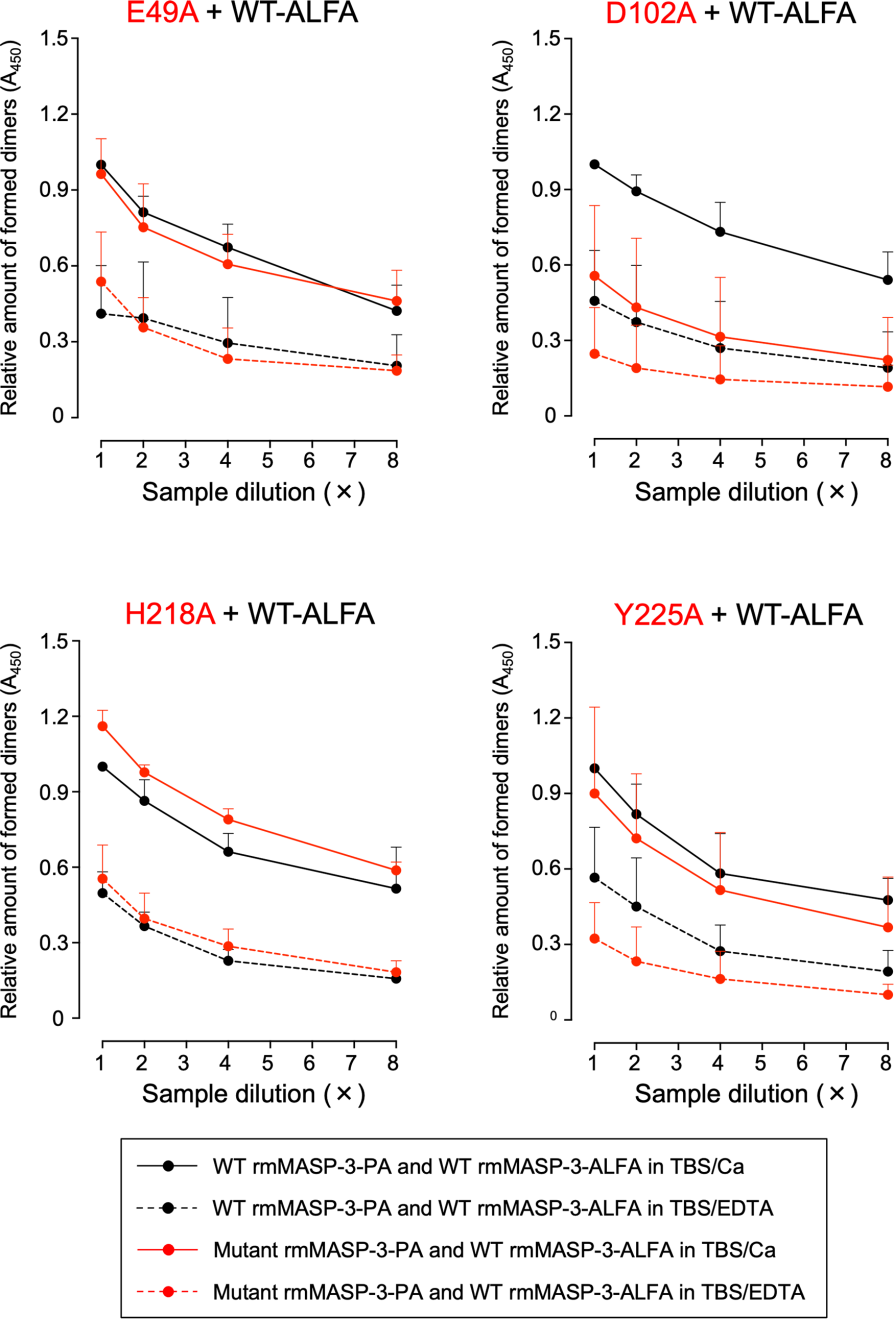
Effects of single amino acid mutations on dimer formation of rmMASP-3-PAs with WT rmMASP-3-ALFA. The black circles represent the formed dimer of WT rmMASP-3-ALFA with WT rmMASP-3-PA, and the red circles represent the formed dimer of WT rmMASP-3-ALFA with mutant rmMASP-3-PAs. The solid lines represent the results on protein mixture dialyzed in TBS/Ca for dimer formation, and the dotted lines represent the results on protein mixture dialyzed in TBS/EDTA for dimer dissociation. Values are means + SD (*n* = 3) of the ratio of absorbance at 450 nm of each sample against the absorbance at 450 nm of the top dilution of the “WT-PA and WT-ALFA in TBS/Ca” sample, which were measured in the same microplate. The values of each sample are obtained by subtracting the absorbance at 450 nm of a control sample without mouse serum from the absorbance at 450 nm of each sample.

MAp44 and sMAP are splicing variants of MASP-1/-3 and MASP-2 transcribed from the *MASP1* and *MASP2* genes, respectively. Both MAp44 and sMAP lack the serine protease domain (L-chain) but have CUB1-EGF-CUB2 domains. Therefore, these proteins, including MASP-1 and MASP-2, are thought to form homodimers or heterodimers with each other *via* the CUB1-EGF-CUB2 domains in their H-chains (26–28). Although dimer formation of MASP-3 has not yet been clarified, it is also considered that MASP-3 forms the homodimers or heterodimers, since MASP-3 has a H-chain common to those of MASP-1 and MAp44. We hypothesized that homodimer formation of MASP-3 is involved in the long-term retention of MASP-3 in the circulation, and faster clearance of the four mutant rmMASP-3-PAs is due to failure of homodimer formation. To clarify this hypothesis, we generated WT rmMASP-3 protein as an ALFA-tag fusion protein, designated WT rmMASP-3-ALFA, and tested whether WT rmMASP-3-ALFA forms dimers with WT or mutant rmMASP-3-PAs based on the method described by Rosbjerg et al. (26). WT rmMASP-3-ALFA was mixed with WT, as well as each mutant rmMASP-3-PA, dialyzed against TBS/EDTA for dimer dissociation, and then dialyzed against TBS/Ca for dimer reassociation composed of rmMASP-3-ALFA and each rmMASP-3-PA. The dimers of rmMASP-3-ALFA and each rmMASP-3-PA in the dialyzed mixture were detected by sandwich ELISA using anti-PA and anti-ALFA antibodies. As shown in **Figure 6**, WT rmMASP-3-ALFA formed dimers with WT rmMASP-3-PA as expected. WT rmMASP-3-ALFA also formed dimers with E49A, H218A or Y225A rmMASP-3-PA at similar levels to WT rmMASP-3-PA. On the other hand, WT rmMASP-3-ALFA showed lower levels of dimer formation with D102A rmMASP-3-PA compared to that with WT or other mutant rmMASP-3-PAs. Nevertheless, there were no significant differences in MASP-3 activation and long-term retention of MASP-3 in the circulation between the four mutant rmMASP-3-PAs, suggesting that homodimer formation of MASP-3 was not involved in the long-term retention of MASP-3 in the circulation. Importantly, the results of the present study suggest that mouse MASP-3 forms homodimers, and D102 in the CUB1 domain of mouse MASP-3 may play an important role in its formation.

## Discussion

MASP-1 and MASP-2 can be activated under certain conditions without forming a complex with LP-PRMs (29, 30). However, the activation efficiency of MASP-1 and MASP-2 is maximized when LP-PRMs complexed with them bind to their ligands. This activation mechanism gave rise to our initial hypothesis that complex formation of MASP-3 with LP-PRMs is involved in MASP-3 activation, at least with regard to its efficiency. To verify our hypothesis, we generated WT and mutant rmMASP-3-PA proteins, the latter of which showed significantly reduced complex formation with MBL-A, MBL-C, ficolin-A, and CL-K1, although it is unclear whether the mutant rmMASP-3-PAs have ability of forming a complex with CL-L1 or CL-LK. Then, we tested their activation kinetics *via* an *in-vivo* experiment. The results presented in this report indicate that the impaired MASP-3/LP-PRM complex formation had little-to-no effect on MASP-3 activation, or its efficiency; however, the results manifest a faster clearance of mutant rmMASP-3-PAs from the circulation compared with WT rmMASP-3-PA.

Since MASP-3 is a serine protease that professionally cleaves pro-FD of the AP, it can be regarded as a complement factor of the AP. On the other hand, like MASP-1 and MASP-2, MASP-3 forms a complex with PRMs of the LP. The current study indicates that, unlike MASP-1 and MASP-2, complex formation of MASP-3 with LP-PRMs is not required for MASP-3 activation or its efficiency. There arises a question as to what the significance of complex formation of MASP-3 with LP-PRMs in the complement system is. When MASP-3 was first discovered, it was known to have a serine protease domain in the L-chain of MASP-3, but the substrate for MASP-3 was unknown (10). Regarding the role of MASP-3 in the complement system, MASP-3 was thought to be a regulator of LP activation because it competes with MASP-2 for complex formation with LP-PRMs (10). Later, MAp44 and sMAP, which form a complex with LP-PRMs but lack the serine protease domain, were found to play a regulatory role in LP activation (27, 31). In 2010, a major turning point came in the research of MASP-3. Takahashi et al. generated *Masp1* gene-knockout mice that were deficient for both MASP-1 and MASP-3, and demonstrated that these mice lacked both LP and AP activity with a proenzyme form of FD in their circulation (16, 32). While in humans, it was reported that the sera of 3MC syndrome patients deficient for both MASP-1 and MASP-3 lacked C4 deposition activity on mannan *via* the LP but exhibited lowered hemolytic activity *via* the AP (33, 34), suggesting the existence of a backup system that activates pro-FD at least in humans. Oroszlán et al. reported that MASP-3 is present predominantly in an active form in human resting blood, and proposed an intracomplex activation mechanism of MASP-3 *via* a proenzyme form of MASP-1 (8). These reports suggested potential crosstalk between the LP and AP *via* intracomplex activation mechanism. To clarify the individual roles of MASP-1 and MASP-3 in the LP and AP, our group generated mice that were monospecifically deficient for MASP-1 or MASP-3 and found that the MASP-1-deficient mouse sera lacked LP activity alone, whereas the MASP-3-deficient mouse sera lacked AP activity alone and had a proenzyme form of FD. In addition, MASP-3 and FD were detected predominantly as active forms in the MASP-1-deficient mouse sera. Taken together, these results indicate that MASP-1 and MASP-3 play independent roles in LP and AP activation, respectively. In other words, there is no crosstalk between the LP and AP *via* intracomplex activation mechanism of MASP-3 by MASP-1. Recently, Oroszlán *et al.* demonstrated that proprotein convertase subtilisin/kexin (PCSK) 5 and PCSK6 are able to activate MASP-3 in resting blood, and concluded that PCSK6 present in sera and plasma is likely to be a major MASP-3 activator in the circulation (35). This report provided important insights into the activation mechanism of MASP-3. However, the role and importance of MASP-3 forming a complex with LP-PRMs remained unclear. In the current study, we demonstrated that WT rmMASP-3-PA, which showed a complex formation with LP-PRMs, remained in the circulation longer than four mutant rmMASP-3-PAs, which showed an impaired complex formation with LP-PRMs. Therefore, we concluded that complex formation of MASP-3 with LP-PRMs may contribute to the long-term retention of the circulating MASP-3.

We next investigated whether the impaired complex formation of MASP-3 with LP-PRMs has an impact on the complement system. We tested restoration of the active FD and AP activity in MASP-3-deficient mice by using sera 3 h after intravenous administration of WT and mutant rmMASP-3-PAs. As a result, all MASP-3-deficient mice showed the same level of active FD and AP activity restoration as WT mice, regardless of whether the administered rmMASP-3-PA has high ability of forming a complex with LP-PRMs (**Figure 5**). These results indicate that the impaired complex formation of MASP-3 with LP-PRMs does not impact restoration of active FD and AP activity in MASP-3-deficient mouse sera at least 3 h after administration. These results also suggest that free MASP-3, without having formed a complex with LP-PRMs, plays predominant roles in the cleavage of the pro-FD and maintenance of AP activity, rather than MASP-3 in a complex with LP-PRMs. However, the latter MASP-3 may act as a reservoir to maintain AP activity, since the proenzyme rmMASP-3-PA, which has high ability of forming a complex with LP-PRMs, can remain longer in the circulation (**Figure 4**).

As described above, the current study showed that WT rmMASP-3-PA can stay longer in the circulation than mutant rmMASP-3-PAs, which have a single amino acid substitution for alanine in the CUB1-EGF-CUB2 domain at E49 (E49A), D102 (D102A), H218 (H218A) or Y225 (Y225A). We observed that the mutant rmMASP-3-PAs showed significantly reduced complex formation with LP-PRMs in the circulation. We hypothesized that these mutant rmMASP-3-PAs may be also unable to form heterodimers or homodimers, which may lead to rapid clearance of rmMASP-3-PA from the circulation. To determine this possibility, we conducted *in-vitro* experiments to test whether these mutant rmMASP-3-PAs can form homodimers with WT rmMASP-3-ALFA. We found that all mutant rmMASP-3-PAs, except mutant rmMASP-3-PA with the D102A mutation, formed homodimers with WT rmMASP-3-ALFA at the same level as homodimer formation between WT rmMASP-3-PA and WT rmMASP-3-ALFA. From these results, we concluded that homodimer formation of rmMASP-3-PA may not contribute to the long-term retention of rmMASP-3-PA in the circulation. Of note, rmMASP-3-PA with the D102A mutation showed decreased homodimer formation with WT rmMASP-3-ALFA. This finding suggests that D102 of MASP-3, a common amino acid sequence between the transcripts of the *Masp1* and *Masp2* genes (i.e., MASP-1, MASP-2, MASP-3, MAp44, and sMAP), may play an important role in the formation of homodimers and/or heterodimers.

In the current study, impaired complex formation of MASP-3 with LP-PRMs showed no significant impact on the complement system, at least in AP activity. On the other hand, lack of MASP-3 activity due to mutations in MASP-3-specific exon of the *MASP1* gene results in 3MC syndrome (20). Of interest, the genes *COLEC10* and *COLEC11*, which transcribe the LP-PRMs CL-L1 and CL-K1, respectively, are also reported to be mutated in the 3MC families (21, 22). In the process of embryonic development, it has been suggested that MASP-3 complexed with CL-K1 functions as a guidance cue for neural crest cell migration (21). Furthermore, CL-L1 and CL-K1 were found to form the heteromeric complex CL-LK, which can form a complex with MASPs including MASP-3 (13). Therefore, the main role of the MASP-3/LP-PRM complexes, especially the MASP-3/CL-LK complex, may be in embryonic development rather than in host defense.

We were unable to determine whether mutant rmMASP-3-PAs formed a complex with CL-L1 or CL-LK. However, we consider that complex formation of rmMASP-3-PA with CL-L1 or CL-LK may not significantly affect the retention of circulating rmMASP-3-PA because of the following reasons. Human serum level of CL-L1 is 0.306 µg/mL and significantly lower than those of MBL (1 µg/mL) or ficolins (ficolin-2: 3.3–5.0 µg/mL, ficolin-3: 18.4–32.6 µg/mL) (36). Furthermore, previous studies have reported that ficolin-3 is the most predominant LP-PRM forming a complex with MASP-3, compared to ficolin-2 and MBL (11). Based on these previous reports, it is likely that the MASP-3 in complex with CL-L1 or CL-LK accounts for a significantly lower proportion of the circulating MASP-3/LP-PRM complexes also in mice. Therefore, it is unlikely that the complex formation of mutant rmMASP-3-PAs with CL-L1 or CL-LK significantly affect the overall *in-vivo* clearance kinetics of MASP-3.

In conclusion, our results demonstrate that complex formation of MASP-3 with LP-PRMs is not required for activation of MASP-3 or its efficiency. However, our results also show that the MASP-3/LP-PRM complexes may contribute to the long-term retention of MASP-3 in the circulation. Our study is the first to show *in-vivo* kinetics of MASP-3 demonstrating rapid activation and clearance in the circulation. Our study also demonstrated that free MASP-3 alone plays a sufficient role in restoring active FD and AP activity in MASP-3-deficient mice. To date, abnormal control of the AP has been shown to exacerbate the pathology of complement-related inflammatory diseases such as age-related macular degeneration (AMD) and kidney disease including atypical hemolytic-uremic syndrome (aHUS), C3 glomerulonephritis, dense-deposit disease, and lupus nephritis (37–39). In these diseases, the development of anti-complement agents that inhibit activation of the AP is eagerly awaited, and MASP-3, which is located upstream of the cascade reaction of the AP, is a promising target. Although further studies are needed, the results of the current study may provide useful information for the development of therapeutic agents targeting MASP-3.

## Data availability statement

The original contributions presented in the study are included in the article/supplementary material. Further inquiries can be directed to the corresponding author.

## Ethics statement

The animal study was reviewed and approved by The Animal Experiments Committee of Fukushima Medical University.

## Author contributions

KK and TM are equally credited as first authors in this work. KK, TM, and HS designed the study and wrote the manuscript. KK, TM, and YI performed the experiments, analyzed the results, and made figures and tables. TO and TS assisted with the experiments. MS and IW designed and assisted with the experiments. TM, TF, and HS supervised the study. All authors contributed to the article and approved the submitted version.

## Funding

This entire research was supported by JSPS KAKENHI Grant Numbers JP19K07610, JP21K07083, and JP22K07122.

## Conflict of interest

The authors declare that the research was conducted in the absence of any commercial or financial relationships that could be construed as a potential conflict of interest.

## Publisher’s note

All claims expressed in this article are solely those of the authors and do not necessarily represent those of their affiliated organizations, or those of the publisher, the editors and the reviewers. Any product that may be evaluated in this article, or claim that may be made by its manufacturer, is not guaranteed or endorsed by the publisher.
